# Emerging Therapies in Pulmonary Fibrosis: A Common Pathway for the Action of Diverse Pharmacologic Agents

**DOI:** 10.3390/ijms27219777

**Published:** 2026-10-30

**Authors:** Graeme B. Bolger

**Affiliations:** BZI Pharma LLC, 100 Cummings Center, Suite 241-D, Beverly, MA 01915, USA; graemebolger@bzipharma.com or gbbolger@uab.edu

**Keywords:** nerandomilast, treprostinil, PDE4 phosphodiesterases, cAMP, extra-cellular matrix, fibroblasts

## Abstract

Fibrotic lung diseases are a heterogeneous set of disorders characterized by progressive infiltration of the pulmonary interstitium by inflammatory cells and fibroblasts, leading to destruction of normal pulmonary tissue and increased extracellular matrix deposition. Fibrotic lung disease can be initiated by numerous triggers—genetic, immunologic, pharmacologic, environmental, infectious—that share many downstream effectors, leading to injury patterns with substantial pathologic and clinical overlap. Recent preclinical studies have expanded our knowledge of the mechanisms of lung inflammation and implicated the key role of specific cytokines in the pathogenesis of lung inflammation. They reinforce the concept that inflammation is a key precursor of lung fibrosis. They have also identified specific intracellular signaling pathways that are modulated, at least in part, by cytokine signaling and that have been shown to be emerging targets for drugs that target inflammation in the lung. Recent advances in therapeutics have yielded two new drugs active in pulmonary fibrosis, the PDE4-selective inhibitor nerandomilast and the prostacyclin analog treprostinil. There is now compelling evidence that, despite their structural differences, these two drugs act through a common mechanism, the cAMP signaling pathway. The new clinical trial results have provided critical confirmation that these targets are indeed key regulators of lung fibrosis and inflammation and, in turn, have provided well-validated clinical evidence that these agents have therapeutic value in patients.

## 1. Introduction

Fibrotic diseases of the lung are a heterogeneous group of disorders that, until recently, have received less scientific and clinical attention than other lung diseases, such as chronic obstructive pulmonary disease (COPD) and pulmonary arterial hypertension (PAH). Recently completed clinical trials of two new drugs, nerandomilast and treprostinil, that offer meaningful clinical benefit to patients with these disorders have drawn increased attention to the spectrum of fibrotic lung disease and offered new options for treatment. In turn, data obtained from these clinical trials, and the pre-clinical evaluation of these new drugs, and those in late-stage clinical trials, has provided new insight into the pathophysiology of these disorders.

This review will first focus on the definition and classification of the spectrum of fibrotic lung disorders, with special emphasis on how advances in the pathophysiology of these disorders have led to improved definitions of these diseases. It will then review recent advances in the immunopathology of these disorders, with special emphasis on how various disease triggers lead to the activation of common mechanisms of immune-mediated lung injury. Emphasis will be placed on key intracellular signaling pathways that mediate immune attack on lung tissue, leading to fibroblast activation and fibrosis. It will then turn to the pre-clinical and clinical-trial data on the two new therapies for fibrotic lung disease, with special emphasis on how these data clarify our knowledge of pro-inflammatory mechanisms and processes in the lung. Finally, the review will conclude by identifying new priorities for both basic and clinical research.

## 2. Interstitial Lung Disease, Idiopathic Pulmonary Fibrosis (IPF), and Progressive Pulmonary Fibrosis (PPF): Overlapping but Distinct Disorders

The term interstitial lung disease (ILD) includes a broad spectrum of lung disorders that are characterized by abnormalities in tissue surrounding alveoli (i.e., the pulmonary interstitium) that have the potential to impair the interchange of oxygen and carbon dioxide between alveoli and pulmonary capillaries [1]. The spectrum of ILD is closely related to, and overlaps with, restrictive lung diseases, which are defined according to spirometry, with reduced lung volumes, impaired lung expansion (inelasticity) and increased stiffness (reduced elastic recoil). Restrictive lung disease, in turn, can be differentiated from obstructive lung disease (e.g., chronic obstructive pulmonary disease, or COPD; ref. [2]) on the basis of spirometry, clinical features, and response to therapy. Pulmonary fibrosis is a subset of ILD defined by fibroblast proliferation in the alveolar interstitium that produces progressive pulmonary dysfunction [1,3,4]. Idiopathic pulmonary fibrosis (IPF) is defined as a fibrotic lung disorder that is associated with usual interstitial pneumonia (UIP); it is not typically associated with an underlying systemic medical condition and arises from inflammation and/or fibrosis that is confined to the lung [5,6]. Progressive pulmonary fibrosis (PPF) is defined as a condition arising in a patient with ILD of known or unknown etiology other than IPF who has radiological evidence of pulmonary fibrosis and clinical, spirometric or radiological evidence of disease progression [6,7].

There are several well-defined triggers for pulmonary fibrosis that provide key insights into its pathogenesis. The genetics of fibrotic lung disorders is an active area of investigation, and there are well-validated associations between the development of IPF and germline variants (single nucleotide polymorphisms, or SNPs) in the telomerase genes *TERT* and *RTEL1*, with emerging evidence for the involvement of other loci involved in DNA metabolism or integrity [8,9,10,11]. SNPs within the promoter region of the *MUC5B* gene, which encodes a protein component of mucin, are associated with the development of ILD in patients with rheumatoid arthritis [12,13,14]. Systemic auto-immune disorders that produce PPF include scleroderma (synonym: systemic sclerosis), rheumatoid arthritis, hypersensitivity pneumonitis, and several other rheumatologic disorders (Table 1; refs. [15,16,17,18,19]). Fibrotic diseases of the lung can develop after certain viral infections, such as COVID-19 [20]. Exposure to any one of several drugs, including bleomycin, several other anti-neoplastic agents, and amiodarone, has been firmly associated with ILD and fibrotic lung disease [21,22]. Closely related to IPF and PPF are fibrotic lung diseases associated with exposure to particulates, including asbestosis [23] and silicosis [24]. Although there is substantial clinical and pathogenic heterogeneity among these disorders, it is likely that there is substantial overlap between their pathogenesis. Advances in our knowledge of the pathophysiology of these disorders have expanded the list of agents available for their treatment [6].

## 3. Global Definition of Mechanisms and Targets in Pulmonary Fibrosis: Insights from Single-Cell Sequencing and Proteomics

Recent advances in single-cell RNA sequencing and proteomics have the potential to greatly expand and clarify the pathogenic mechanisms of fibrotic lung disease and thereby accelerate the pre-clinical development of agents that target these fundamental processes. Among the most startling conclusions from the single-cell sequencing studies has been the discovery of hitherto-unappreciated cell types in the human lung [31,32,33,34,35]. These studies have provided compelling evidence for the existence of up to 58 cell types in normal murine and human lung [33,34], many of which are not apparent on routine histopathologic examination of lung tissues. They have served to complement and expand histopathological studies on lung tissue from patients with COPD and pulmonary fibrosis [36,37,38], yielding new insights into the pathophysiology of these disorders. Single-cell sequencing studies have also provided valuable insights into commonly used experimental models for pulmonary fibrosis [39,40,41], as described in more detail below. The RNA-seq data have been confirmed and expanded by proteomic studies, such as a very recent analysis of protein profiles in 58 human tissues, including the lung, by data-independent mass spectrometry [42] that provides essential information on the cellular distribution of known and potentially future drug targets.

## 4. Fundamental Pathways in the Pathogenesis of Pulmonary Fibrosis: Key to Effective Therapies

### 4.1. Pathway 1: cAMP Signaling

Signal transduction mediated by the canonical small molecule “second messenger” 3′, 5′ cyclic adenosine monophosphate (cyclic AMP or cAMP) plays an essential role in numerous organs and tissues, including the lung, the endocrine system, inflammation and immunity, and the CNS (Figure 1). The synthesis of cAMP by membrane-associated adenylyl cyclase is regulated, at least in part, by a number of key G-protein-coupled receptors (GPCRs). GPCRs are targets for small molecules or peptides that can act as physiological mediators, hormones, neurotransmitters, or growth factors. Among the most important GPCRs in the lung is the β_2_-adrenergic receptor (β_2_-AR), the target for β-adrenergic agonists in airway smooth muscle and other lung tissues [2]. Prostaglandin agonists also modulate cAMP signaling through GPCRs, as described in detail below. Also key to the regulation of cAMP signaling in cells are cyclic nucleotide phosphodiesterases (PDEs) that hydrolyze (degrade) cAMP (and/or 3′, 5′ cyclic guanosine monophosphate [cGMP]) and thereby modulate its levels in cells [43,44,45,46,47]. cAMP exerts its downstream effects by binding to, and regulating the activity of, several effector proteins, including the cAMP-dependent protein kinase (PKA), cyclic-nucleotide-gated ion channels, exchange protein activated by cAMP (EPACs) and popeye proteins [48,49,50,51,52,53,54,55]. The regulation of cAMP synthesis and hydrolysis is tightly controlled in both space and time, producing “compartments”, “gradients” or “pools” that are responsible for its highly specific downstream effects.

### 4.2. Fundamental Implications of cAMP Signaling in the Pathogenesis and Management of Pulmonary Fibrosis

Extensive pre-clinical and clinical data have provided extensive support for the involvement of cAMP signaling pathways in pulmonary fibrosis, as described in detail in subsequent sections. Specifically, members of the cAMP-specific phosphodiesterases, or PDE4 family, are the targets for the action of roflumilast and nerandomilast, both of which have significant action in patients with pulmonary fibrosis. Similarly, the IP receptors, which are GPCRs that modulate cAMP signaling, are the major targets for the action of treprostinil and related drugs, which also have clinical benefit in many patients with pulmonary fibrosis. The similarities in cellular action of these agents (Figure 1), and the extensive pre-clinical data on these agents, provide convincing support for the key role of cAMP signaling in pulmonary fibrosis. We will discuss the key pre-clinical data in subsequent sections; following that discussion, we will review the results of pivotal clinical trials.

### 4.3. Pathway 2: Receptor Tyrosine Kinase Signaling

Among the most important regulators of fibroblast proliferation and differentiation are signals generated by the action of transmembrane receptor tyrosine kinases (RTKs; Figure 2). These RTKs serve as the receptors for numerous peptides that regulate growth and development, including vascular endothelial growth factor (VEGF; refs. [56,57] platelet-derived growth factor (PDGF; ref. [58]), and basic fibroblast growth factor (FGF; ref. [59]). These three growth factors stimulate directly the proliferation of fibroblasts, as well as initiating developmental cascades that eventually transform dividing fibroblasts into fibrocytes and regulate the production of extracellular matrix proteins. In addition, RTK signaling stimulated by these growth factors acts co-operatively with other signal transduction pathways, most notably those mediated by cytokines and other inflammatory mediators. In this way, RTK-mediated signal transduction can act additively or synergistically with inflammation-mediated signaling to generate the final fibrotic phenotype. We will discuss this concept in greater detail below, especially in relation to pre-clinical models of fibrotic lung disease.

### 4.4. Pathway 3: Transforming Growth Factor—Beta (TGF-β) Signaling

TGF-β is a member of a large class of peptide growth factors and has been implicated in numerous cellular processes, including the cell cycle, cell growth and differentiation, and the response to injury [60]. The mechanisms of TGF-β action are complex, but many of its actions are exerted through a canonical pathway where TGF-β dimers bind to the extra-cellular domain of the TGF-β Type II receptor, which then recruits, phosphorylates and heterodimerizes with the Type I receptor to produce an active signaling complex (Figure 3). The activated Type I receptor then phosphorylates a member of the SMAD family of signaling proteins (canonically, SMAD2 and SMAD3), which then bind with SMAD4 and translocate to the nucleus. The trimeric SMAD2/3/4 complex interacts with several families of transcription factors to regulate the transcription of specific target genes. The TGF-β receptor can also signal through several non-SMAD signaling pathways, including the RAS/RAF/MAPK signaling pathway, the PI3K/AKT/mTOR pathway (Figure 2) and the JAK/STAT3 pathway, all of which can regulate transcription. Signaling through the TGF-β pathway, especially through SMAD2/3, has been shown to be a critical regulator of tissue fibrosis in diverse tissues, notably the kidney, liver, and heart [61,62,63,64]. TGF-β signaling has also been implicated in a number of pre-clinical models of fibrotic lung disease, as described in detail below.

### 4.5. Pathway 4: Tumor Necrosis Factor α (TNFα) Signaling

TNF is a pro-inflammatory cytokine that is released from activated macrophages and other inflammatory cells. It acts on numerous populations of immune and inflammatory cells, where, depending on the cellular context, it can stimulate proliferation and maturation, or produce apoptosis [65,66,67]. TNF regulates numerous signal transduction pathways in inflammatory cells but exerts many of its immune/inflammatory effects through its regulation of the NF-κB pathway (Figure 4). TNF can also act directly on fibroblasts, where it has pro-proliferative and differentiating effects, as described in the sections below. Other TNF receptor superfamily receptors may also contribute to fibrosis development, such as the p75 neurotrophin receptor, which is regulated, at least in part, by its interaction with the PDE4 family member PDE4A4/5 [68].

### 4.6. Emerging Pathways

In addition to the well-understood cAMP and growth-factor-RTK signaling pathways, several other regulatory processes have been implicated in fibroblast activation and therefore in the mechanisms of fibrotic lung disease.

#### 4.6.1. Fibroblast Activation Protein (FAP)

FAP was first characterized in the context of the tumor microenvironment [69]. It is an antigen on the surface of activated fibroblasts, especially cancer-associated fibroblasts, with a structure reminiscent of a receptor: it has a large extracellular domain with serine protease activity, a transmembrane region, and a short cytoplasmic “tail”. The extracellular domain has dipeptidyl peptidase and endopeptidase activities, targeting extracellular matrix proteins, especially collagen types I and III. Through its peptidase activity, FAP also has the ability to cleave and activate α2-antiplasmin and to cleave and inactivate FGF21. These biochemical functions implicate FAP in growth factor signaling and the regulation of extracellular matrix production. FAP expression can be activated in several contexts, including tissue healing, inflammation and oncogenesis. Its role in pulmonary fibrosis has yet to be determined precisely, but it is an excellent candidate to mediate interactions between inflammatory/immune/allergic processes and the development of fibrosis as a response to these stimuli.

#### 4.6.2. Serotonin Receptors

Serotonin, a ubiquitous neurotransmitter with numerous effects both in and outside of the brain, acts through seven distinct receptor families (5-HT1 through 5-HT7), six of which are GPCRs [70,71]. The 5-HT3 receptor family is a ligand-gated ion channel. Serotoninergic metabolites have been implicated in the fibrotic cardiac valve disease associated with exposure to serotonergic drugs, such as fenfluramine [70]. Signaling through cardiac adenosine receptors, another set of GPCRs, may contribute to fibrosis in the myocardium [72]. Intriguingly, the cardiac valve pathology does not appear to be associated with a profound inflammatory response. 5-HT receptors have been shown to be present in honeycomb cysts and fibroblast foci, suggesting their potential role as a target for therapy in pulmonary fibrosis [73]. Emerging evidence supports a role for serotonin signaling in certain animal models of fibrotic lung disease, as discussed in detail below.

## 5. Inhibitors of cAMP-Specific (PDE4) Phosphodiesterases and Agonists of the Prostacyclin IP Receptor Have a Common Downstream Action

Two important drugs used in the therapy of pulmonary fibrotic disease are nerandomilast and treprostinil. Both have been the subject of recently reported clinical trials (Section 7) and have been approved, or are undergoing review, for clinical use in IPF and/or PPF by regulatory authorities in several geographies, including the USA and the EU. Nerandomilast acts specifically as an inhibitor of the cAMP-specific, or PDE4, family of cyclic nucleotide phosphodiesterases, while treprostinil acts specifically as a prostacyclin analog, as described in more detail below. Among the most important insights from the pre-clinical and clinical data on nerandomilast and treprostinil is that they have a common downstream action: they increase cAMP levels in cells and thereby activate cAMP signaling (Figure 1). To underscore the importance of this insight, we will review the biology of these two components of the cAMP signaling pathway.

### 5.1. The PDE4s, or cAMP-Specific Phosphodiesterases, and Pulmonary Fibrosis

The cAMP-specific phosphodiesterases, or PDE4 enzymes, are major contributors to the regulation of cAMP signaling in diverse cell types. The PDE4s are members of the cyclic nucleotide phosphodiesterase superfamily, which is divided into 11 families, encoded by a total of 21 genes [43,44,46,74]. The individual members of the PDE superfamily are defined by their substrate specificity and their ability to be inhibited by pharmacologic inhibitors specific to each family. PDE3, PDE4, PDE7 and PDE8 are cAMP-selective, PDE5, PDE6 and PDE9 are cGMP-selective, while PDE1, PDE2, PDE10 and PDE11 are dual-specific. The substrate specificity and the ability to be selectively inhibited by family-specific inhibitors are determined by the primary amino acid sequence and fold of the catalytic regions of each family member(s) [75,76]. Therefore, PDE-selective inhibitors act, at least in part, at the catalytic sites of the PDE enzymes and therefore act, at least in part, as competitive inhibitors of cAMP and/or cGMP hydrolysis. By inhibiting the hydrolysis of cAMP, they increase cAMP levels in cells and thereby increase the activity of PKA and other substrates of cAMP action (Figure 1).

Understanding the role of PDE4 inhibitors lung disease requires an appreciation of the incredible diversity of PDE4 isoforms. There are over 20 PDE4 isoforms, which are encoded by 4 genes in mammals (*PDE4A*, *PDE4B*, *PDE4C* and *PDE4D* in humans), with additional diversity being produced by alternative mRNA splicing and the use of promoters specific for each isoform [44,77,78]. Specifically, the human *PDE4B* gene encodes 5 different isoforms [79] and *PDE4D* encodes 9 different isoforms [80]. Each isoform has a distinct pattern of expression in tissues, suggesting that each has a distinct cell, tissue or organismal function. Given the complexity of cell types and gene expression in each of these cell types (as described above), the patterns of expression of each PDE4 isoform in lung are just beginning to be determined.

Although PDE4-selective inhibitors have been available for decades [77,78], only recently has it been possible to generate compounds that are relatively selective for isoforms encoded by each of the four PDE4 genes. For example, roflumilast and its active metabolite, roflumilast N-oxide, are highly selective for PDE4 (as opposed to other PDE families), but inhibit all PDE4 isoforms relatively equally (i.e., IC_50_ values that vary from 0.2 to 7.8 nM, depending on the PDE4 isoform, ref. [81]). In contrast, nerandomilast shows selectivity for PDE4B (IC_50_ values of 10 nM for PDE4B, 91 nM for PDE4D, 248 nM for PDE4A, and 8700 nM for PDE4C, ref. [82]). Although further analysis of the isoform specificity of nerandomilast would be very helpful (see ref. [83] for a detailed discussion), the success of this drug in the clinic provides encouragement for continued development of PDE4-selective inhibitors that are highly isoform-specific and also have other highly desirable drug qualities. To date, PDE4 is the only PDE family member to be a pharmacologically relevant target in pulmonary fibrosis, as explained in more detail below. In contrast, PDE5 inhibitors modulate nitric oxide—cGMP signaling and have been shown to be safe and effective in the treatment of PAH [84,85].

### 5.2. Prostacyclin Analogs and Pulmonary Fibrosis

Among the most important regulators of cAMP signaling in the lung, and certainly one of the best-understood pathways implicated in the pathogenesis of pulmonary fibrosis, is signaling through the receptors for prostaglandins and their metabolites [86,87,88]. At least 11 different GPCRs serve as receptors for various members of the prostaglandin family: PGE2 signals through the EP1, EP2, EP3, and/or EP4 receptors, PGD2 through the DP1 receptor, and prostacyclin (PGI2) through the IP receptor. The EP2, EP4, DP1, and IP GPCRs activate adenylyl cyclase (Figure 1), increase cAMP levels in cells, and are felt to have an anti-fibrotic effect [88]. In contrast, the EP1, EP3, and DP2 GPCRs work through several different G proteins to inhibit adenylyl cyclase. PGD2 also signals through the CRTH2 receptor on γδT-cells, which in turn regulates the release of IL-10, which has an anti-fibrotic effect [89]. In contrast, PGF2α acts through the FPA and FPB GPCRs to stimulate PKC and thereby produce a pro-proliferative, pro-fibrotic phenotype. Additionally, platelet-derived thromboxane A2 (TXA2) acts through the TBXA2 receptor (which exists in two forms, TPα and TPβ, both GPCRs) that, depending on the cellular context, can act through Gs to stimulate adenylyl cyclase, or act through Gi to inhibit adenylyl cyclase, ultimately exerting a pro-fibrotic effect [88]. The TXA2 receptors also act through Gq proteins to trigger calcium signaling pathways.

Given the diversity of prostaglandin receptor types, there would appear to be multiple modes of interaction between prostaglandin signaling molecules and intracellular signaling pathways that involve cAMP signaling (Figure 1). To date, pulmonary fibrosis researchers have focused their attention largely on prostacyclin. Prostacyclin signals through the IP receptor, a GPCR, which stimulates adenylyl cyclase (see preceding paragraph and Figure 1) and has potent vasodilatory action on the pulmonary arterial vasculature. Therapeutically, prostacyclin analogs, such as treprostinil, are mainstays in the treatment of PAH, which is felt to be mediated by their action on the IP receptor [90]. In the treatment of pulmonary fibrosis, some of the effect of treprostinil is likely to be mediated by the IP receptor, although emerging evidence suggests that some of its effects are mediated through other prostaglandin receptors (Section 6.1 and Section 6.2). Other prostaglandin family members, including thromboxane, which acts through a set of receptors that is very different from that of the IP receptor (see preceding paragraph), also appear to have antifibrotic effects [91].

### 5.3. cAMP Signaling: A Common Mechanism of Action for PDE4-Selective Inhibitors and Prostacyclin Analogs

Given that both PDE4-selective inhibitors, such as nerandomilast and roflumilast, and prostacyclin analogs, such as treprostinil, elevate cAMP levels in cells, and thereby activate downstream signaling through PKA and other cAMP downstream effectors (Figure 1), it is reasonable to predict that they might act additively or even synergistically in pre-clinical models, and potentially in humans. Evidence for the cooperative action of PDE4-selective inhibitors, prostacyclin analogs, and prostaglandins has been demonstrated in a variety of pre-clinical models of fibrosis, as described in detail in the sections below. These studies provide additional confirmation that these two different sets of compounds can act together through their modulation of cAMP signaling, despite their lack of structural similarity.

## 6. Insights from Pre-Clinical Models of Lung Fibrosis

### 6.1. Inflammation as a Precipitant of Fibrotic Lung Disease

Inflammation has been recognized universally as a major, if not the major, contributor to pulmonary fibrosis in humans. Both PDE4-selective inhibitors and prostaglandin analogs have extensive effects on inflammation and immunity.

#### 6.1.1. PDE4 Inhibition and Inflammation

Many, potentially all, of the actions of PDE4-selective inhibitors in pulmonary fibrosis can be traced to their anti-inflammatory and immunomodulatory actions. First-generation PDE4 inhibitors have long been known to have anti-inflammatory effects in pre-clinical models of asthma and COPD (see ref. [78] for a review of older, but still-relevant data, and ref. [44] for recent updates). The anti-inflammatory effects of roflumilast—and its active metabolite, roflumilast N-oxide—are mediated through its action on numerous cell types, including CD4+ and CD8+ T-cells, monocytes/macrophages, and neutrophils [81]. Roflumilast also has potent effects on airway and vascular smooth muscle and respiratory epithelium. It has been shown to prevent or ameliorate pulmonary hypertension in several rodent models, and potentially in humans, which is likely to reflect a direct action on vascular smooth muscle cells [81]. Roflumilast is a potent stimulator of the cystic fibrosis transmembrane regulator (CFTR) Cl− ion channel, essential for mucociliary clearance and the maintenance of airway surface liquid (see ref. [92] for a review). Although any of the immune and non-immune effects of roflumilast could contribute to its beneficial effects in patients with pulmonary fibrosis, its anti-inflammatory effects are probably paramount. The pre-clinical data on nerandomilast continue to emerge, but its effects on immune/inflammatory cells are broadly similar to those of roflumilast [82,93,94,95]. The effects of roflumilast and nerandomilast on several different mouse models of inflammatory disease that lead to lung fibrosis are discussed below.

#### 6.1.2. Prostaglandins and Inflammation

Prostaglandins and their metabolites have long been shown to play an essential role in various aspects of inflammation, notably in the triggering of pain and fever, as well as mediating vascular responses to inflammatory stimuli. Various members of the prostaglandin family, especially PGE2, have also been shown to have direct effects on immune cells, including dendritic cells, macrophages, and T cells [88,96,97,98]. In the lung, PGE2 modulates myriad processes, including the actions of neutrophils and eosinophils, the production of numerous cytokines, the differentiation of Th1 and Th2 cells, and has direct effects on airway and vascular smooth muscle [86,87].

### 6.2. PDE4 Inhibitors, Prostaglandins and Prostacyclin Analogs All Have Direct Effects on Fibroblast Proliferation and Activity in the Lung

Numerous pre-clinical studies have demonstrated that roflumilast and nerandomilast have substantial anti-fibrotic effects. Studies on human and rodent fibroblast preparations have shown that roflumilast and other PDE4-selective inhibitors directly attenuate the actions of IL-1 on FGF-induced proliferation of human fetal lung fibroblasts [99,100,101]. When combined with the β_2_-adrenergic receptor agonist indacaterol, roflumilast significantly inhibited the release of the pro-fibrotic factor endothelin-1 and the production of extracellular matrix proteins [102]. Nerandomilast has been shown to induce the dedifferentiation of human fibroblast precursors from patients with IPF and to diminish their contractility in vitro [93].

The major action of PGE2 in pulmonary fibrosis appears to be the inhibition of fibroblast proliferation/migration and reduced synthesis of extracellular matrix [103,104], compatible with an anti-fibrotic effect. Consistent with this conclusion, reduced PGE2 action is a hallmark of scleroderma [15].

PGE2 analogs act additively or synergistically with PDE4 inhibitors in diverse fibroblast models, including three-dimensional collagen gels [105], human fibroblast preparations [100,104,106,107,108,109,110], and an extracellular matrix model [111]. The cooperative effects of PDE4 inhibitors and PGE2 analogs on cell-based models have been confirmed and expanded by research using animal models of fibrotic lung disease, as discussed in detail in the next section.

Treprostinil acts in the lung primarily as a prostacyclin analog (i.e., on the IP receptor), which accounts for its vasodilatory effects. However, emerging pre-clinical data have provided evidence for its action at other GPCRs, including EP2 and DP1, as well as the PPARβ receptor, which may account for many of its anti-fibrotic effects [90]. Alternatively, the antifibrotic effects of treprostinil may be mediated by its action on the IP receptor [112]. The effects of treprostinil on mouse models of inflammatory disease that lead to fibrosis are discussed below.

### 6.3. Effects of PDE4 Inhibitors, Prostaglandins, and Prostacyclin Analogs in Animal Models of Fibrotic Lung Disease

#### 6.3.1. Genetic Models of Fibrotic Lung Disease

Given the well-characterized human germline mutations that strongly predispose to fibrotic disease, including in the lung, it would be of considerable interest to develop rodent genetic models that reproduce essential features of human fibrotic lung disease. To date, genetically modified mice, mostly notably *Tert* knockouts, have not been useful as models of human genetic fibrotic disorders, although they have been essential models of aging, senescence and tumorigenesis [113,114].

#### 6.3.2. Models of Silicosis and Asbestosis

Chronic exposure to the particulates asbestos and silica produces fibrotic lung disease in humans that shares many features with IPF. Chronic treatment of mice with silica produces lung inflammation and fibrosis that resembles human silicosis [24,115,116] and which can be alleviated with treatment with pirfenidone [24] or nerandomilast [82,116]. To date, a convincing animal model of asbestosis has yet to be developed.

#### 6.3.3. Radiation-Induced Pulmonary Fibrosis

Radiation-induced tissue injury is an important dose-limiting toxicity of radiation therapy for cancer and other conditions in humans and produces changes in lung tissue that histologically and clinically overlap with other human fibrotic lung diseases. Radiation produces fibrotic changes in mouse lung that resemble human radiation-induced lung disease [117,118]. Radiation-induced lung fibrosis in mice is accompanied by abnormalities in prostaglandin signaling pathways, especially for thromboxane A, suggesting a role for TBXA2 receptor antagonists as therapy for pulmonary fibrosis [91].

#### 6.3.4. Murine Inflammatory Disease Models

Inflammatory myopathies are chronic immune disorders that can affect numerous organ systems and are associated with characteristic auto-antibodies [30]. Several clinical and immunologic features are predictive of the development of ILD in patients with inflammatory myopathies [119]. The expression of PDE4B is up-regulated in lung tissue from humans with inflammatory myopathy and treatment with nerandomilast reduces markers of inflammation and fibrosis in these patients [94]. Nerandomilast treatment of mouse models of ILD associated with inflammatory myopathies improves lung fibrosis scores and downregulates a number of key signaling pathways leading to lung fibrosis [94,95].

#### 6.3.5. Bleomycin-Induced Pulmonary Inflammation and Fibrosis

Bleomycin-induced pulmonary fibrosis is among the most widely employed murine models of lung fibrosis. Bleomycin is an anti-neoplastic agent used widely in several human cancers; it is a potent generator of free radicals, which accounts for both its antitumor activity and its toxic effects on normal tissues. Intratracheal treatment of mice with bleomycin produces an intense inflammatory reaction rich in macrophages, neutrophils and lymphocytes. Over several weeks, this inflammatory reaction progresses to severe fibrosis. Single-cell sequencing, proteomic, and high-dimensional histologic studies in mice with bleomycin-induced lung injury have demonstrated a step-wise transformation from inflammation to fibrosis, with subsequent recovery, that is associated with the development of specific fibroblast subtypes that develop in response to pro-fibrotic and anti-fibrotic factors [120]. The progression of bleomycin-induced lung inflammation is attenuated by corticosteroids, which also prevent or ameliorate the resulting fibrosis [21]. In contrast, tobacco smoke typically produces lung inflammation that does not progress to fibrosis and which does not respond to glucocorticoids [81].

Numerous mediators of inflammation and fibroblast proliferation/development have been implicated in the pathogenesis of bleomycin-induced pulmonary fibrosis. Elevated levels of TNF-α, TGF-β, several interleukins, including IL-13, and other cytokines are present in bronchoalveolar lavage and lung tissue from mice treated with bleomycin, especially in the early inflammatory stage, and are felt to be a major contributor to the development of lung inflammation and subsequent fibrosis [121,122,123,124,125]. In contrast, serotonin signaling may protect against bleomycin-induced inflammation, in that mice with knockouts of *Tph1* (which encodes tryptophan hydroxylase I, the rate-limiting enzyme in serotonin synthesis), develop significantly higher levels of inflammation and fibrosis when treated with bleomycin [126]. Mice with a deletion of the CRTH2 receptor, which serves as a PGD2 receptor on γδT-cells, developed higher levels of inflammation and fibrosis upon bleomycin treatment, supporting a role for this T-cell subset in the inflammatory response [89].

All the oral drugs that are currently approved for the treatment of human pulmonary fibrosis show activity in the bleomycin-induced fibrosis mouse model. Pirfenidone reduces inflammation in this model, and its effects are correlated with lower expression of TGF-β and pro-collagen gene expression [127,128]. Nintedanib also has been shown to have significant activity in this model, reducing inflammation and the expression of many cytokines and other mediators of inflammation [129,130,131,132,133,134,135,136]. Roflumilast and the PDE4-selective inhibitor cilomilast have been tested extensively in this model, with substantial efficacy [81,121,122,137]. Nerandomilast has also been tested in this model and shown to be effective, with immune and cellular effects that are similar to those shown previously with other PDE4-selective inhibitors [82]. Inhibition of 15-prostaglandin dehydrogenase (15-PGDH), the key enzyme in the degradation of PGE2, increases PGE2 levels in the lung and reduces the severity of bleomycin-induced lung injury in mice [138]. Treprostinil also has activity in the bleomycin-induced fibrosis mouse model [139,140], although the precise mechanism of its action (i.e., IP receptor v EP1/2 receptors) in this model remains to be determined.

## 7. Insights from Clinical Trials

### 7.1. Clinical Trials of Pirfenidone and Nintedanib

Pirfenidone was the first agent to be shown on the basis of multiple phase 3 clinical trials to be safe and effective in IPF [18,141,142,143,144]. It has also been shown in observational trials to have some benefit in other fibrotic disorders of the lung, including asbestosis [23] and bleomycin-induced pulmonary toxicity [21]. The pharmacologic target of pirfenidone remains uncertain, even after considerable research; it is clearly not a PDE4-selective inhibitor or prostaglandin analog [145,146,147]. Nintedanib also has been shown on the basis of multiple phase 3 clinical trials to be safe and effective in both IPF and PPF [16,148,149,150,151,152]. Nintedanib targets several protein-tyrosine kinases, most convincingly the receptors for vascular endothelial growth factor (VEGF), platelet-derived growth factor (PDGF), and fibroblast growth factor (FGF, Figure 3; see also the pre-clinical studies section, above). Combination therapy with both of these agents appears to have additive benefit [151,153,154,155].

### 7.2. Clinical Trials of PDE4-Selective Inhibitors

Several phase 3 clinical trials have shown substantial clinical benefit of PDE4-selective inhibitors in COPD, especially in patients with a bronchitic phenotype (reviewed in [83,92]). PDE4-selective inhibitors have also shown to be effective in other inflammatory disorders, most notably eczema, atopic dermatitis, psoriasis and psoriatic arthritis [83,156]. The PDE4B-selective inhibitor nerandomilast has been shown on the basis of two phase 3 trials to be safe and effective in both IPF and PPF [157,158,159,160,161]. The data from these and related clinical trials show that nerandomilast clearly improves symptoms and leads to improvement in several key clinical measures, although a survival benefit was uncertain [160,162,163,164,165]. The magnitude of benefit seen with nerandomilast in these studies appears to be at least as good as that seen with pirfenidone or nintedanib, although this conclusion currently reflects cross-trial comparisons; more definitive conclusions will require head-to-head comparisons in clinical trials. In one of the two phase 3 clinical trials of nerandomilast [158], toxicities were as follows: For doses of 18 mg, 9 mg, and placebo, events leading to discontinuation of trial regimen (all causes) occurred in 10.0%, 8.1% and 10.2%, respectively, over a 52-week period. Diarrhea occurred in 36.6%, 29.5% and 24.7% of patients and was a reason for discontinuation of therapy in 2.6%, 1.3%, and 0.5% of patients, respectively. Diarrhea appeared to be more prevalent in patients also taking nintedanib, being a reason for discontinuation in 4.1%, 2.9%, and 1.2% of patients, respectively, compared to 1.4%, 0.0% and 0.0%, respectively, for those not taking nintedanib. Nausea occurred in 10.2%, 7.6%, and 6.6% of each group, respectively. Weight loss occurred in 10%, 9% and 8% of each group, respectively. Finally, depression occurred in 10.2%, 9.7% and 11.2% of each group, respectively [158,159]. In the second phase 3 clinical trial, for doses of 18 mg, 9 mg, and placebo, diarrhea occurred in 41%, 31%, and 16% of patients and was reason for discontinuation of therapy in 6%, 2% and 1% of patients, respectively [159]. Although no formal statistical analyses were performed on these toxicity data, reflecting current practice, these phase 3 clinical trial data demonstrate that the GI toxicities (typically nausea, diarrhea and weight loss) of nerandomilast are possibly lower than those of older PDE4-selective inhibitors, such as rolipram, cilomilast or roflumilast, although this conclusion currently reflects cross-trial comparisons; more definitive conclusions will require head-to-head comparisons in clinical trials. Potential pharmacologic reasons for the benefits of nerandomilast have been discussed elsewhere [83].

The toxicities of nerandomilast identified in these two phase 3 clinical trials were of special interest in view of those experienced in prior clinical studies of PDE4-selective inhibitors (i.e., the class-defining side effect profile). The authors conclude that “adverse events of interest given that nerandomilast inhibits PDE4, such as vasculitis, depression, and suicidality, were balanced across trial groups” [159]. Other class-defining side effects, such as GI toxicity and weight loss, have been described above. Any comparison of the toxicity or efficacy of nerandomilast to older PDE4-selective inhibitors, such as roflumilast, would of course require a head-to-head comparison in a phase 3 clinical trial.

### 7.3. Clinical Trials of Prostacyclin Agonists

The synthesis and breakdown of prostaglandins, thromboxane, and their precursors have been extensive subjects for pharmacologic investigation. Here, we will focus on clinical trials of prostaglandins and agonists of their cognate GPCRs in pulmonary arterial hypertension (PAH) and pulmonary fibrosis. Agonists of the IP receptor (i.e., prostacyclin analogs) have been studied extensively in PAH. PAH can develop in the absence of any other underlying lung disease (i.e., primary PAH), or can develop as a secondary manifestation of COPD, ILD, IPF, or PPF. Initial studies of IP receptor agonists focused on short-lived agents that required intravenous infusions, and in a few cases, direct instillation into the pulmonary artery [166]. Subsequent clinical trials focused on inhaled prostacyclin analogs, such as iloprost [167,168]; see ref. [85] for a review. More recent clinical trials for prostacyclin analogs in PAH have focused on treprostinil, which can be administered in two different inhaled forms (nebulization or dry powder inhaler), as well as intravenously and orally. Initial phase 3 clinical trials focusing on PAH-ILD showed that treprostinil improved symptoms, but a potential survival advantage was uncertain [169,170,171,172,173]. A phase 3 clinical trial of nebulized treprostinil for PAH-COPD was terminated early for an increase in serious adverse events and suggestive evidence of an increased risk of mortality [174]. Very recently, two phase 3 clinical trials of nebulized treprostinil in patients with IPF without clinical evidence of PAH showed improvement in pulmonary function tests and fewer clinical-worsening events compared to placebo; the studies lacked statistical power for detecting an improvement in survival [175,176]. Toxicities with treprostinil were provided in a pooled analysis from both trials. Events leading to discontinuation of trial regimen (all causes) occurred in 18.8% of patients, compared to 13.6% with placebo, with cough, dyspnea and headache as the major contributors [175]. Diarrhea occurred in 16.4% of patients with treprostinil and 21.1% with placebo (with 95% confidence intervals overlapping) and was a reason for discontinuation of therapy in 0.7% of patients in both treprostinil and placebo arms [175]. Collectively, these clinical trials provide convincing evidence for the therapeutic value of treprostinil in fibrotic lung diseases and provide considerable confirmation of the insights obtained from the pre-clinical models described above.

### 7.4. Ongoing and Planned Clinical Trials

A search of www.clinicaltrials.gov on 30 August 2026 using the search term “nerandomilast” and no filters revealed a total of 36 clinical trials that included nerandomilast, of which 24 were completed, 7 were actively recruiting, and 5 had not yet opened (see also the compilation in [177]). A search for treprostinil (using the drug name as search term, no filter, all dosage forms) yielded 154 studies, of which 18 were recruiting, and 6 had not yet opened. The majority of the ongoing trials were extensions of the pivotal trials described above, or were studying either agent in new populations (e.g., pediatric patients, or specific inflammatory disorders). The results of the ongoing and planned studies are awaited with interest, as they have the potential to provide further confirmation of the pre-clinical data described above.

Given that nerandomilast and treprostinil both act by increasing cAMP levels in cells, it is perhaps surprising that none of the trials listed on Clinicaltrials.gov were studying the combination of nerandomilast and treprostinil as dual therapy. Nerandomilast and inhaled treprostinil have acceptable toxicities as single agents, and these toxicities appear to be largely non-overlapping, despite their common mechanisms of action. Therefore, combination therapy is worth investigating in this patient population, although well-designed clinical trials would be essential to prove safety and efficacy. Concerns about the feasibility and cost of dual therapy would also need to be carefully addressed. Potential concepts for future clinical trials would also include testing of combined therapy (e.g., either nerandomilast or treprostinil in combination with other agents), or of agents still under development.

## 8. Future Directions

Recent advances in pre-clinical knowledge of pharmacologic interventions in fibrotic lung disease, and the results from recent pivotal clinical trials, have greatly accelerated our understanding of its biology and also led to innovations that show great promise in improving the lifespan and quality of life for patients with this disorder. These innovations have sparked a number of important new perspectives on its biology and clinical management (see Box 1, Key Points).

Box 1 **Key Points.**

Key Points:

1. Pulmonary fibrosis is a heterogeneous group of disorders with diverse genetic and environmental triggers.
2. The development of biomarkers that reflect the underlying pathogenesis of this disorder will be useful in defining prognosis, the choice among current therapies, and in stratifying patients enrolled in the next generation of clinical trials.
3. Despite the heterogeneity of this disorder, inflammation-induced proliferation and differentiation of fibroblasts is key to its development.
4. Activation of cAMP signaling in immune and other cells in the lung is a key mechanism of action of two new drugs for this disorder, nerandomilast and treprostinil.

Among the most fundamental advances in pulmonary fibrosis is a greatly increased appreciation of the heterogeneity of this disease. Several key studies have identified patients whose disease reflects a strong genetic predisposition, and further study of patients in biobanks and other genetic-focused registries has the potential to identify further deeply penetrant alleles predisposing to this disorder. These studies also have the potential to identify less-penetrant alleles that interact with environmental factors in the pathogenesis of “sporadic” or “environmental” cases of fibrotic lung disease. Similarly, there appear to be subsets of pulmonary fibrosis that are defined largely by known environmental triggers, such as asbestos or silica. Finally, there is a large subset of patients whose disease reflects an abnormality of the immune system.

Currently, the population of patients with immunologically defined fibrotic lung disease is sub-divided according to the presence or absence of a defined extra-pulmonary immune disorder; however, in the future, it is likely that these categories will be replaced, or at least sub-divided, into groups defined by immunologic (or possibly, genetic or environmental) factors. These immunologic factors will reflect underlying differences in the immunopathogenesis of the disorder in individual patients and will be useful in defining prognosis, the choice among current therapies, and in stratifying patients enrolled into the next generation of clinical trials. The development of biomarkers that reflect the underlying pathogenesis of this disorder, and which reflect its heterogeneity, will be essential in these efforts. Analysis of the clinical and laboratory data generated on the large number of patients that have been enrolled in recently completed clinical trials and registry studies [178] is therefore an important priority.

Despite the heterogeneous nature of pulmonary fibrotic disease, it is of considerable interest that two classes of agents that have been shown clearly to have activity in this disorder have largely overlapping mechanisms of action. Both PDE4-selective inhibitors and prostacyclin analogs exert the vast majority of their pharmacologic effects by increasing the levels of cAMP in cells, and thereby activating downstream effectors of cAMP signaling, such as PKA (Figure 1). It is likely that many of their effects are mediated through CREB-mediated transcription (Figure 1), and further analysis of single-cell sequencing and proteomic data is likely to clarify the gene activation events responsible for their therapeutic effects. In particular, single-cell analyses will aid in elucidating the exact cell populations and tissues that are targets for these agents. It is likely that most of these targets are immune cells, although a direct action of either/both PDE4 inhibitors and/or prostacyclin analogs on fibroblasts or other mesenchymal cells is still a plausible contributor. In contrast, in other lung disorders, such as asthma and certain subsets of COPD, cAMP levels are increased by pharmacologic action at different drug targets/tissues, such as the β_2_-adrenergic receptor in airway smooth muscle (see ref. [92] for a review).

Although targeting immune/inflammatory cell populations through cAMP signaling pathways is likely to be the most important effect of PDE4-selective inhibitors and prostacyclin analogs in pulmonary fibrosis, other agents active in fibrotic lung disorders act directly on fibroblasts and other components of fibrotic tissue (Figure 5). The success of pirfenidone and nintedanib in pulmonary fibrosis, the latter of which appears to target primarily fibroblasts and which clearly acts on pathways other than cAMP signaling (Section 7.1), shows that direct therapeutic targeting of fibroblasts has clear clinical benefit. The results of clinical trials of new agents, as well as well-validated cohort studies [179], have the potential to further define the cellular targets for agents of therapeutic value in this disorder.

Priorities for further pre-clinical study include experiments identifying interactions between the various signaling pathways implicated in the development of fibrosis in the lung (Section 4). It is highly likely that these pathways do not exist in isolation, but interact dynamically in ways that are not currently appreciated. Newly developed approaches, including single-cell sequencing (transcriptomics, Section 3) and phospho-proteomics, have the ability to identify members of each pathway that undergo changes in expression and/or secondary modification (specifically phosphorylation) in response to stimuli that drive another pathway. These studies therefore have the potential to identify “cross-talk” between cAMP signaling pathways and other pathways mediating inflammation or fibrosis.

Although inflammation precedes, and is an essential contributing factor to, the development of fibrosis in the lung, it is entirely possible that inflammation is not essential to the development of fibrosing conditions in other organs and tissues. For example, the development of fibrosis in the cornea, a non-vascularized tissue, is initiated and undergoes progression with minimal evidence of inflammation; instead, fibroblast proliferation and resistance to apoptosis appear to be critical factors [180]. Similarly, the cardiac valvular fibrosis associated with drugs that affect serotonergic signaling occurs with minimal evidence for prior or ongoing inflammation (see references in the description above). Careful comparison of the pathogenesis of fibrosis in diverse organs is likely to provide additional insights into the development of these disorders and may lead to novel therapies.

Although the development of new therapies, especially those with novel mechanisms of action, remains the key objective in research on fibrotic lung disease, additional clinical study of agents that have already been approved for this disorder remains a priority. One obvious area is the testing of combined therapy with treprostinil and nerandomilast, given potential additive or synergistic benefits that would be predicted by their common mechanisms of action. Combining two agents with non-overlapping toxicities, which has already been attempted with the combination of pirfenidone and nintedanib (see Section 7.1 and Section 7.4, above), would also appear to be a priority. Finally, the repurposing of drugs that have been approved previously for indications other than fibrotic lung disease requires further exploration [181].

## Figures and Tables

**Figure 1:**
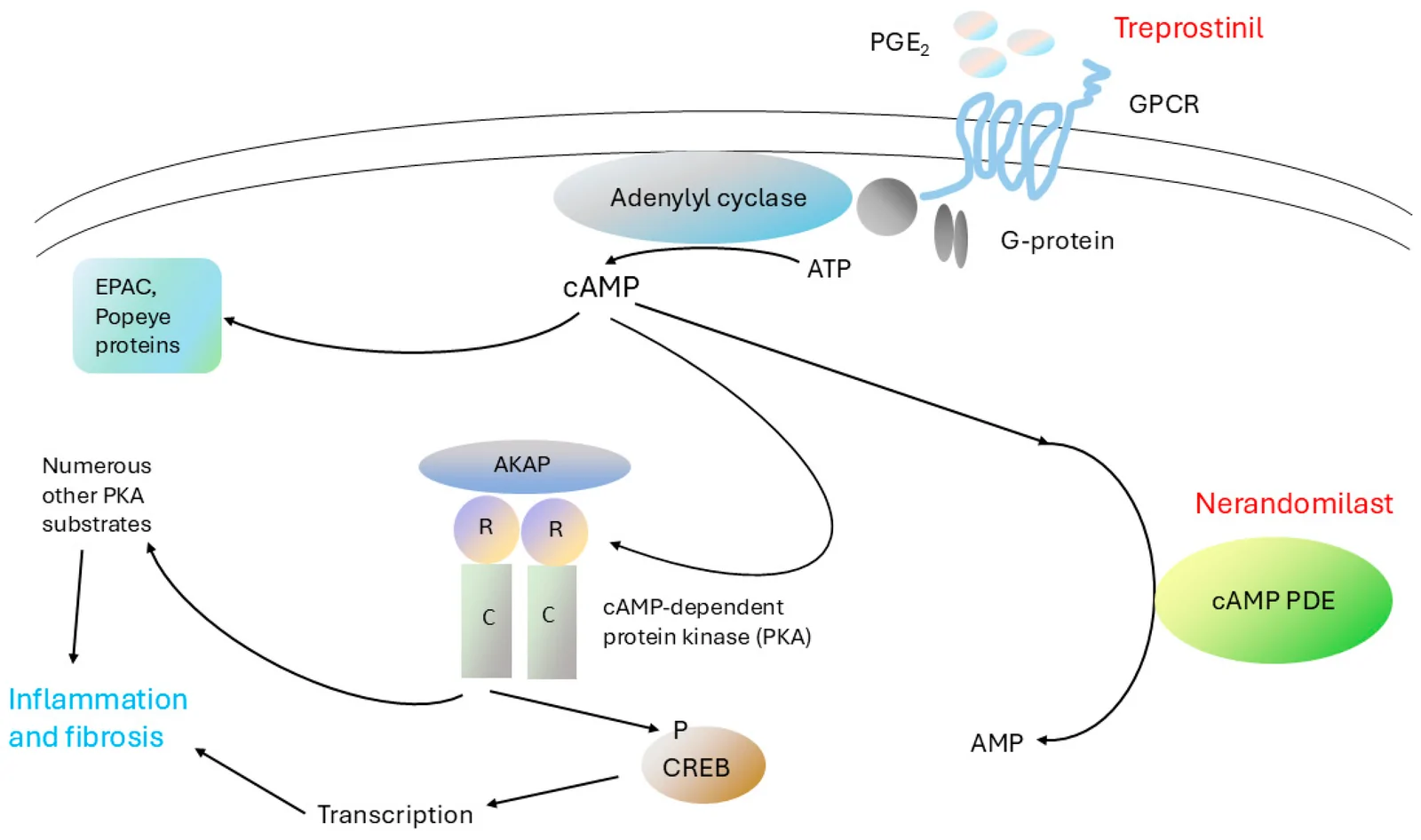
cAMP signaling pathways in inflammatory cells and fibroblasts: Interaction between activation of receptors for prostaglandins and prostacyclin and inhibition of PDE4 cyclic nucleotide phosphodiesterases. G-protein-coupled receptors (GPCRs) comprise a diverse family of 7-helix proteins that span the plasma membrane (represented by the thin curved lines near the top of the figure) and play a pivotal role in transmitting intracellular signals into the cell interior. GPCRs are regulated by small molecules or peptides that bind to their extracellular or transmembrane regions. An important set of GPCRs in pulmonary tissue, inflammatory cells, and fibroblasts are those for prostaglandins, such as PGE2. Treprostinil serves to mimic the action of endogenous prostacyclin by binding to, and activating, the IP receptor, a GPCR. Also important in pulmonary physiology is the β_2_-adrenergic receptor (β_2_-AR), a GPCR that is the target for β-adrenergic agonists. Trimeric G-proteins have three subunits (α, β, and γ) and can be divided into stimulatory (Gsα) or inhibitory (Giα) isoforms by the actions of their α subunits. Activated GPCRs recruit trimeric G-proteins by their ability to bind them to specific regions on their intracellular loops, which in turn activates Gsα and/or the βγ subunits. Activated Gsα stimulates downstream signaling effectors, specifically membrane-associated adenylyl cyclase (AC) isoforms, which catalyze the synthesis of 3′, 5′ cyclic adenosine monophosphate (cAMP). cAMP, the prototypical “second messenger,” can diffuse widely in cells, but in many contexts its concentration is tightly regulated within specific sub-cellular compartments. Important regulators of cAMP signaling include the cAMP PDEs, notably members of the cAMP-specific PDE, or PDE4 family, which selectively hydrolyze cAMP and thereby regulate its levels and action in cells. Nerandomilast and other PDE4-selective inhibitors therefore act to increase cAMP levels in cells. Important downstream targets of cAMP include the cAMP-dependent protein kinase (PKA), the exchange protein activated by cAMP (EPAC), and Popeye proteins. Subcellular compartmentalization of cAMP signaling is often mediated by the anchoring of PKA to specific subcellular locations, through its binding to A-kinase anchoring proteins (AKAPs). PKA is activated by the binding of cAMP to its regulatory (R) subunits; this in turn activates the PKA catalytic (C) subunits, which phosphorylate (P) specific serines and threonines present in diverse PKA substrate proteins. A major PKA substrate is the cyclic nucleotide response-element binding protein (CREB), a loop–helix–loop transcription factor. CREB is activated by phosphorylation by PKA and other kinases, which causes it to dimerize and translocate to the nucleus. Activated CREB binds to specific cyclic AMP response elements in active chromatin, thereby regulating the transcription of numerous genes, including those involved in the modulation of immunity, inflammation and fibrosis.

**Figure 2:**
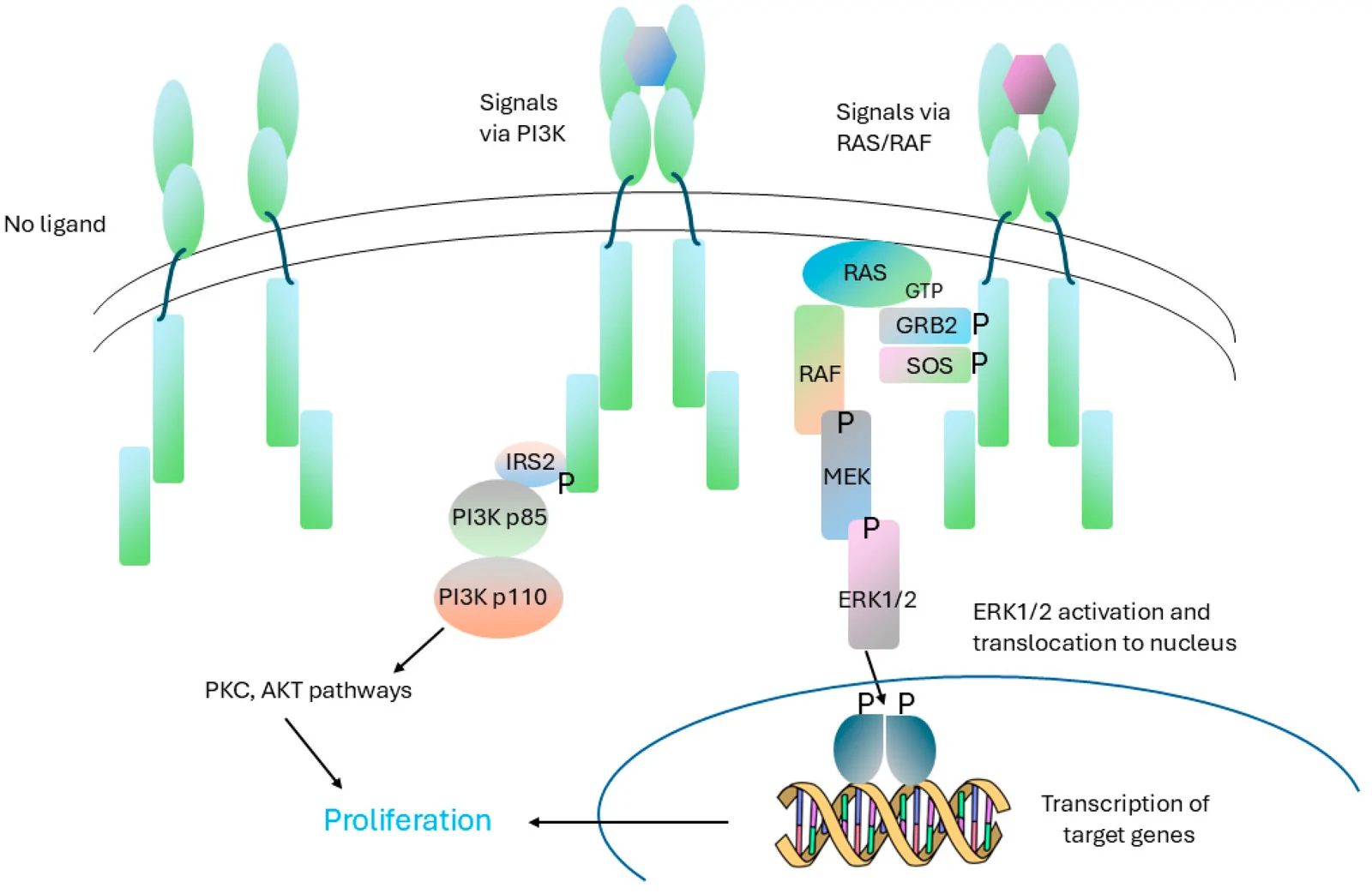
Signaling mediated by receptor tyrosine kinases leading to fibroblast proliferation. Key growth factors, including platelet-derived growth factor (PDGF), fibroblast growth factor (FGF) and vascular endothelial growth factor (VEGF), are released by immune/inflammatory cells, activated fibroblasts, other dividing cells, or platelets. These growth factors act through receptor tyrosine kinases (RTKs) to stimulate fibroblast proliferation and differentiation. Epidermal growth factor (EGF) and other growth factors active on pulmonary epithelium also act on RTKs on fibroblasts. A typical transmembrane tyrosine kinase receptor consists of one or more extracellular domains, a transmembrane region, and one or more intracellular domains, which have the ability to phosphorylate tyrosines on selected substrate proteins. In the absence of ligand, RTKs typically exist as monomers. The binding of ligand (hexagons) to RTK extracellular domains causes them to dimerize, which serves to activate their tyrosine kinase activity. Activated receptors then associate with various inner-membrane-associated and cytoplasmic proteins that in turn feed into downstream signaling pathways. For example, activation of certain RTKs can recruit and phosphorylate the insulin-receptor substrate-2 (IRS-2) protein. IRS-2 acts as an adaptor/scaffold protein for the p85 subunit of phosphoinositol-3-kinase (PI3K p85), which in turn regulates the PI3K catalytic subunit (PI3K p110). PI3K is a serine kinase that can phosphorylate and regulate the activity of a number of downstream signaling proteins, including protein kinase C (PKC) and AKT kinase. Other tyrosine kinase downstream targets include members of the STAT family), transcription factors that regulate the expression of numerous genes involved in differentiation and cell survival. Activation of still other RTKs results in the recruitment and tyrosine phosphorylation of the adaptor proteins GRB2 and SOS, which in turn can recruit and activate the oncogene proteins RAS and RAF. Activation of RAF initiates a serine-threonine kinase cascade, consisting of the MEK and ERK1/2 kinases. Phosphorylation of the ERK1/2 kinases leads to their dimerization and translocation to the nucleus, where they can in turn phosphorylate several classes of loop–helix–loop transcription factors. These activated, phosphorylated transcription factors in turn regulate the transcription of numerous genes involved in cellular proliferation and differentiation. Collectively, the activation of the RTKs therefore serves to stimulate fibroblast division and differentiation.

**Figure 3:**
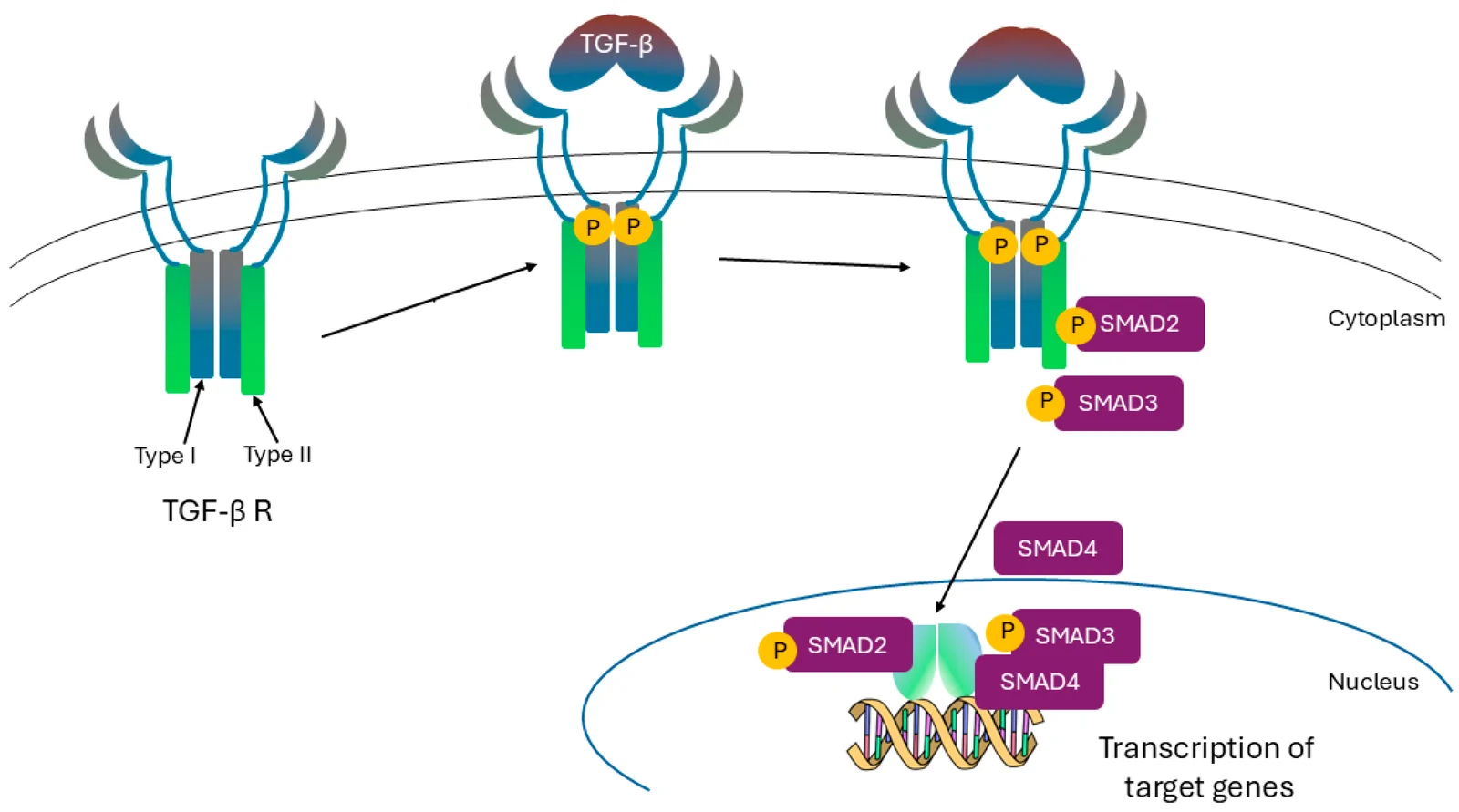
Signaling through the TGF-β receptor and SMADs. The TGF-β receptor (TGF-β R) is a transmembrane receptor with Type I and Type II subunits. Binding of the TGF-β dimer to the Type II receptor stimulates its serine–threonine kinase activity, producing phosphorylation of the Type I receptor and association of two Type I and two Type II receptors into an activated receptor complex. The activated receptor binds and phosphorylates two SMAD family members, SMAD2 and/or SMAD3. Phospho-SMAD2 and phospho-SMAD3 migrate to the nucleus; SMAD4 accompanies the nuclear import of SMAD2 and SMAD3. In the nucleus, phospho-SMAD2 and phospho-SMAD3 associate with transcription factors (green dimer, bound to DNA) and serve to recruit elements of the transcription machinery to regulate the transcription of specific target genes.

**Figure 4:**
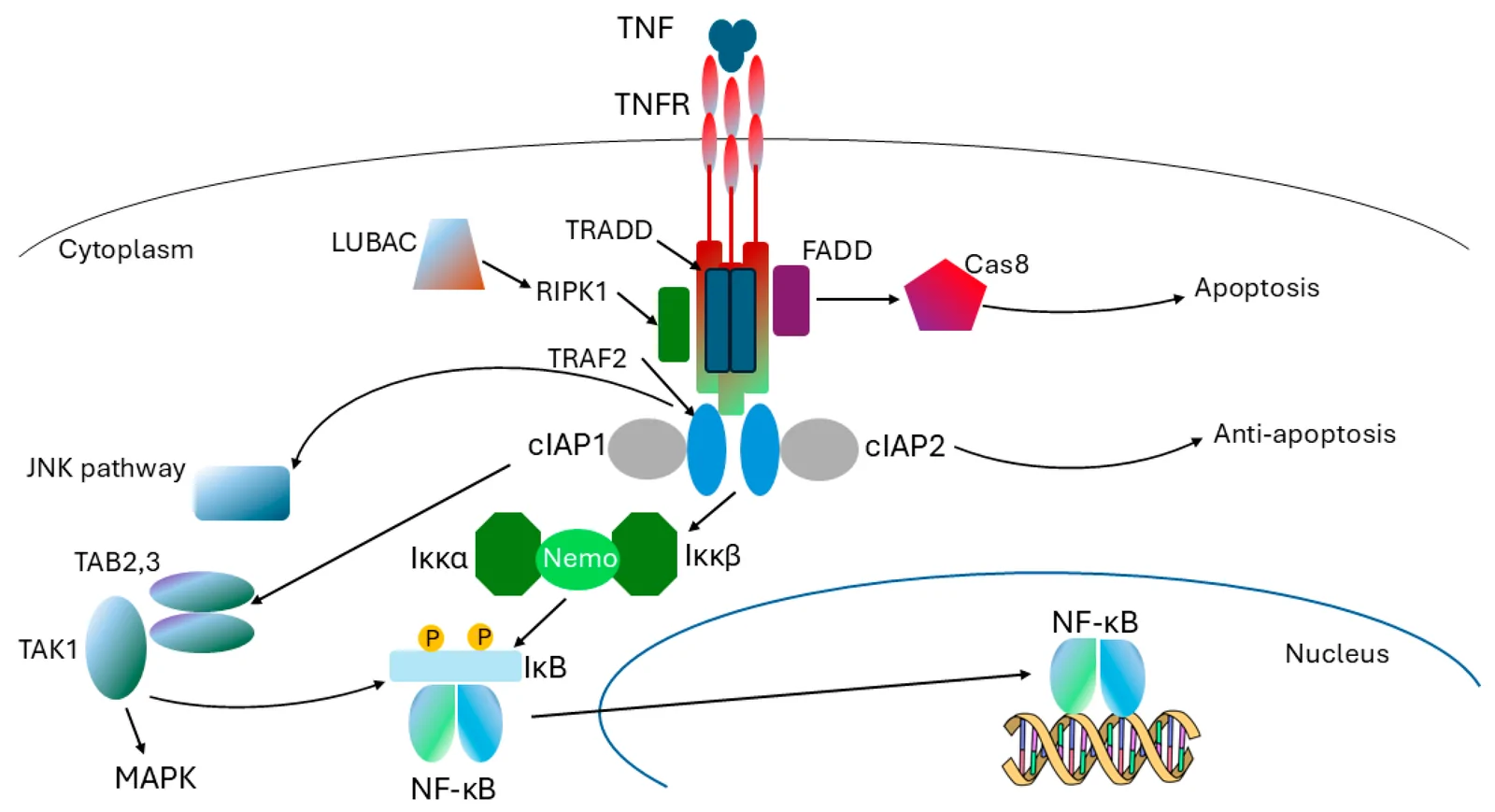
Signaling through the TNF receptor. Tumor necrosis factor (TNF), a key inflammatory mediator, is secreted by macrophages in response to a variety of stimuli. It binds to the extracellular domain of its receptor (TNFR) as a trimer. Binding of TNF to the TNFR causes the dissociation of the inhibitory silencer of death domains (SODD;) protein from the cytoplasmic domain of the TNFR, causing it to associate as a trimer. Activated, ligand-associated TNFR associates with the adaptor protein TNF-receptor associated death domain (TRADD) protein, which works through a second adaptor, the receptor-interacting protein (RIPK1), to recruit other members of the receptor complex. The Fas-associated death domain (FADD) protein recruits caspase-8 (Cas8), thereby activating Cas8 by stimulating its intrinsic self-cleaving activity. Activated Cas8 activates a classic protease cascade that leads to apoptosis. TNFR-associated factor 2 (TRAF2) protein recruits the cellular inhibitors of apoptosis proteins (cIAP1 and cIAP2), which have K63-ubiquitin ligase activity and are generally inhibitors of apoptosis. Among the substrates of cIAP1/2 is RIPK1, leading to the recruitment of the linear ubiquitin chain assembly complex (LUBAC), which has M1-linked ubiquitin ligase activity, and TAK1-binding proteins 2 and 3 (TAB2/3). TAB2/3 then recruit transforming growth factor-β-activated kinase 1 (TAK1), leading to activation of the mitogen-activated protein kinase (MAPK) pathway. TRAF2 also activates a member of the mitogen-activated protein kinase kinase kinase 1, (MAP3K1 or MEKK1) or apoptosis signal-regulating kinase 1,(MAP3K5 or ASK1), family leading to activation of the c-Jun NH_2_-terminal kinase (JNK) pathway. Activation of the TNFR, through ubiquitination of RIPK1 by LUBAC, also leads to recruitment of the IKK complex, which consists of IκB kinases α and β (Iκκα and Iκκβ) and their regulatory subunit, NF-κβ essential modulator (Nemo). Through TAK1, this complex phosphorylates Iκκα/β, which then phosphorylates the inhibitor of κB (IκB). Phosphorylated Iκβ in turn undergoes proteasomal degradation, which releases the bound NF-κB heterodimer. The NF-κB dimer then translocates to the nucleus, where it serves as a transcription factor to regulate the transcription of a specific set of immune-regulatory genes. Many of the components of this receptor complex can also be activated, or inhibited, by other cellular signaling pathways or by the general cellular signaling context.

**Figure 5:**
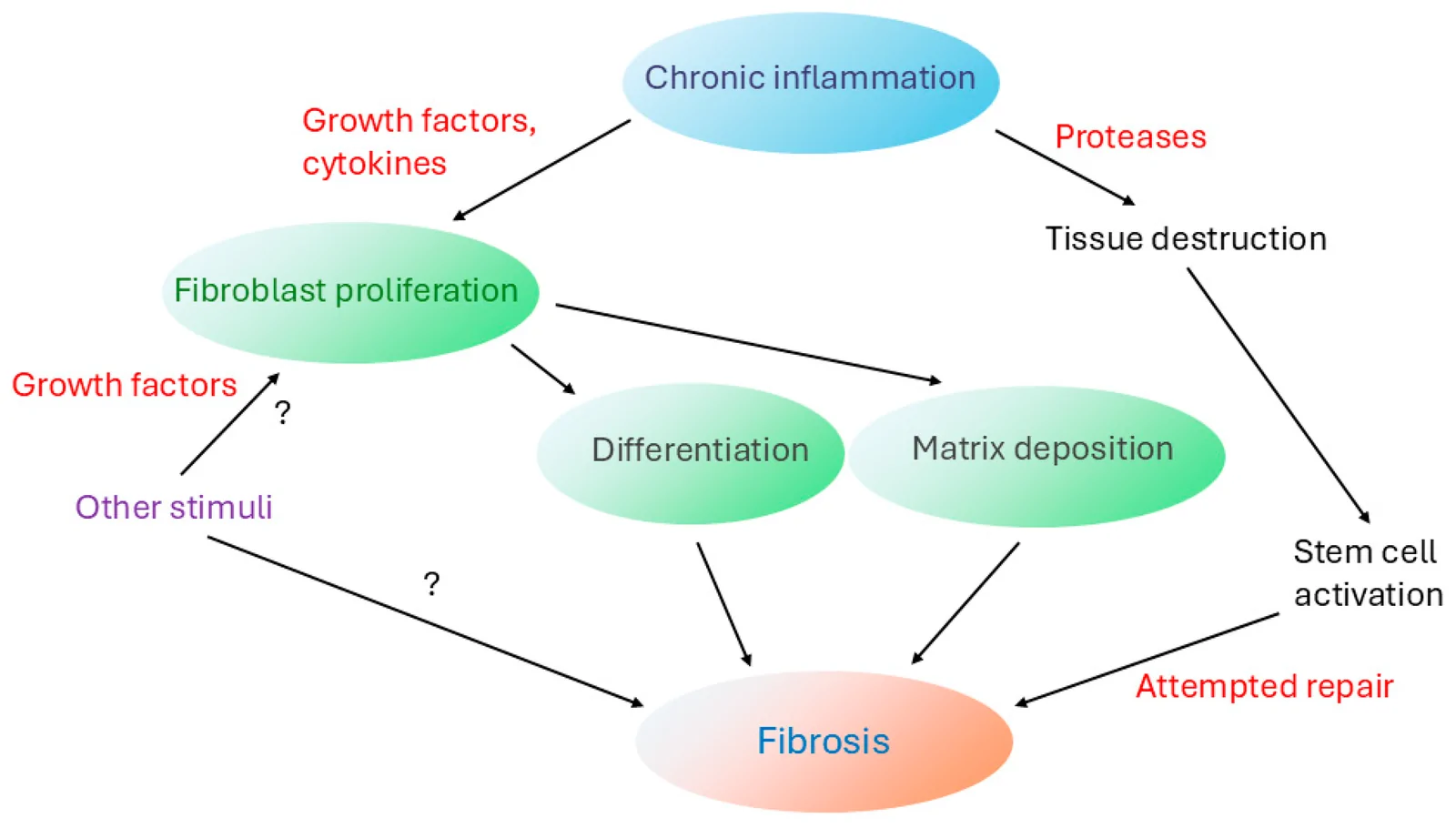
Relationship between inflammation, tissue repair and fibrosis in the lung. Chronic inflammation in the pulmonary interstitium is characterized by the infiltration of activated immune/inflammatory cells, particularly neutrophils, macrophages and lymphocytes. The production and local release of numerous cytokines by inflammatory cells is a key requirement for the proliferation of fibroblasts, their subsequent development into fibrocytes, and the production and deposition of extracellular matrix proteins. Growth factors secreted by inflammatory cells, including PDGF, FGF and VEGF, also serve as potent stimulators of fibroblast proliferation. Proteases produced by neutrophils and other inflammatory cells produce tissue destruction. Attempted repair of damaged pulmonary tissue leads to activation of pulmonary stem cells, which can in turn differentiate into fibrocytes. Other, as yet poorly characterized, stimuli may also contribute to fibroblast proliferation and fibrosis. Pulmonary fibrosis can therefore be viewed as the end effect of multiple events, with chronic inflammation being a key precipitating and maintaining factor. The ? Indicates remaining areas of scientific uncertainty.

**Table 1:** Inflammatory diseases that can lead to the development of pulmonary fibrosis. References: [17,25,26,27,28,29,30].

Disorder	Typical Organ Involvement	Typical Auto-Antibodies
Rheumatoid arthritis	Joints, tendons	Anti-citrullinated proteins
Scleroderma	Skin, mucous membranes	Nuclear proteins, others
Inflammatory myopathies	Skeletal muscle, skin	Splicing factors, tRNA synthetases, others
Hypersensitivity pneumonitis	Airways	Diverse; molds and birds are common sources

## Data Availability

No new data were created or analyzed in this study. Data sharing is not applicable to this article.
